# RETRACTED: Wu et al. Preparation and Analysis of Structured Color Janus Droplets Based on Microfluidic 3D Droplet Printing. *Micromachines* 2023, *14*, 1911

**DOI:** 10.3390/mi15040451

**Published:** 2024-03-28

**Authors:** Chuang Wu, Hanqi Jia, Haithm Yahya Mohammed Almuaalemi, A. S. M. Muhtasim Fuad Sohan, Binfeng Yin

**Affiliations:** 1 School of Mechanical Engineering, Yangzhou University, Yangzhou 225127, China; mz120230875@stu.yzu.edu.cn (H.J.); mh22012@stu.yzu.edu.cn (H.Y.M.A.); 2 School of Electrical and Mechanical Engineering, The University of Adelaide, Adelaide, SA 5005, Australia; asmmuhtasimfuad.sohan@student.adelaide.edu.au

The *Micromachines* Editorial Office retracts the article “Preparation and analysis of structured color Janus droplets based on microfluidic 3D droplet printing” [1], cited above. 

Following publication, the authors raised concerns to the publisher regarding an experiments error that occurred during the initial stages of this study.

Adhering to our complaints procedure, an investigation was conducted by the Editorial Office and Editorial Board that confirmed that the experimental data regarding the impact of the oil phase’s volume ratio on the Janus droplets were erroneously utilized in the subsequent experiments. As a result, the Editorial Office, the Editorial Board, and the authors determined that this error significantly impacted the results of the study and agreed that the overall findings could not be relied upon. Thus, this article has been retracted as per MDPI’s retraction policy (https://www.mdpi.com/ethics#_bookmark30, accessed on 27 February 2024) and in line with the Committee on Publication Ethics retraction guidelines (https://publicationethics.org/retraction-guidelines, accessed on 27 February 2024). 

This retraction was approved by the Editor-in-Chief of the journal *Micromachines*. 

The authors agreed to this retraction.
